# Reconstruction of past distribution for the Mongolian toad, *Strauchbufo raddei* (Anura: Bufonidae) using environmental modeling

**DOI:** 10.7717/peerj.9216

**Published:** 2020-06-05

**Authors:** Spartak N. Litvinchuk, Natalya A. Schepina, Amaël Borzée

**Affiliations:** 1 Institute of Cytology of Russian Academy of Sciences, St. Petersburg, Russia; 2 Biological Department, Dagestan State University, Makhachkala, Russia; 3 Geological Institute, Siberian Branch of Russian Academy of Sciences, Ulan-Ude, Russia; 4 Nanjing Forestry University, Nanjing, China

**Keywords:** Ecological modeling, North East Asia, Refugium, Distribution shift, Holocene, Pleistocene, Toads, Bufonidae

## Abstract

The use of ecological models enables determining the current distribution of species, but also their past distribution when matching climatic conditions are available. In specific cases, they can also be used to determine the likelihood of fossils to belong to the same species—under the hypothesis that all individuals of a species have the same ecological requirements. Here, using environmental modeling, we reconstructed the distribution of the Mongolian toad, *Strauchbufo raddei*, since the Last Glacial Maximum and thus covering the time period between the Late Pleistocene and the Holocene. We found the range of the species to have shifted over time, with the LGM population clustered around the current southern range of the species, before expanding east and north during the Pleistocene, and reaching the current range since the mid-Holocene. Finally, we determined that the ecological conditions during the life-time of the mid-Pleistocene fossils attributed to the species in Europe were too different from the one of the extant species or fossils occurring at the same period in Asia to belong to the same species.

## Introduction

Species distribution models using various environmental factors are gaining in popularity and accuracy. Models have been used to understand ecological requirements of organisms, study niche segregation and facilitate fieldwork by predicting potential occurrence areas, improving conservation, and numerous other applications (e.g., Phillips, Anderson & Schapire, 2006; Litvinchuk et al., 2014; Litvinchuk et al., 2018; Skorinov et al., 2014; Zurell & Engler, 2019). Presence-only records from different sources (scientific papers, museum collections, on-line databases, etc.) provide valuable resources for modeling efforts (Graham et al., 2004). Modeling can also resolve past and future distributions (Sillero & Carretero, 2013; González-Fernández et al., 2018). For instance, the impact of the Quaternary glaciations on biota is a topic of considerable interest and modeling can help answer questions. Thus, environmental modeling allows reconstructing the dynamics of species distribution in the Holocene and Pleistocene (e.g., Garcia-Porta et al., 2012; Wielstra et al., 2013; Skorinov & Litvinchuk, 2016; Dufresnes et al., 2018; Dufresnes et al., 2019; Dufresnes et al., 2020a; Dufresnes et al., 2020b).

The Mongolian toad, *Strauchbufo raddei* (Strauch, 1876), is a widespread amphibian distributed across open landscapes of southern Siberia, the Russian Far East, Mongolia, northeastern China, and northern Korea (Okada, 1935; Szyndlar, 1984; Kim & Han, 2009; Shchepina et al., 2010; Borkin et al., 2011; Dong et al., 2012; Kuzmin, 2013; Fei, Ye & Jiang, 2015; Song, 2016; Kuzmin et al., 2017). The species inhabits various biotopes including forest edge, bushland, meadow, forest-steppe, steppe, semi-desert, and desert (Kuzmin, 2013; Kuzmin et al., 2017). The toad does not avoid anthropogenic and agricultural landscapes. Desert populations live in oases which often are isolated from other populations by vast dry areas.

The fossil records of the Mongolian toad are relatively abundant and widely distributed (e.g., Böhme & Ilg, 2003; Ratnikov, 2009; Shchepina, Kolomiets & Budaev, 2015; Schepina, Khenzykhenova & Namzalova, 2016). Moreover, this is the only modern East-Palearctic anuran species that was previously common both in Asia and Europe. The phylogeography of this species has been studied in detail (Dong et al., 2012). However, no environmental modeling studies have been conducted. High spatial resolution paleoclimate surfaces for global land areas, such as WorldClim (http://www.worldclim.org) and especially PaleoClim (Brown et al., 2018), give opportunity to trace shifts in range boundaries of various animal species during the Pleistocene and the Holocene. The Mongolian toad is a very convenient subject to reconstruct the dynamics of its past distribution using the databases and the modeling’s capabilities.

Here, we have assessed the spatial distribution of *S. raddei* under present environmental conditions with the goal of reconstructing the changes to its distribution during the Pleistocene.

## Materials & Methods

### Current distribution

In Russia *S. raddei* inhabits the southern part of Siberia and the Far East on the territory of Irkutskaya Oblast (25 localities), the Republic of Buryatia (172), Zabaykalsky Krai (31), Amurskaya Oblast (20), Khabarovsky Krai (45), the Jewish Autonomous Oblast (21), and Primorsky Krai (43). An isolated population is found in the southern part of Sakhalin Island (Russia). The species range also covers most of Mongolia (273) with the exception of the western regions. The toad is widespread in the northwestern part of China (74) and the northernmost part of North Korea (9). *S. raddei* was recorded at various altitudes, from sea level up to 3,100 m a.s.l. (in average 733 m, SD = 504 m; Table S1). The highest locality was in Haibei Tibetan Autonomous Prefecture (36°57 ′N, 100°53′E) near Qinghai (or Kuku-Nor) Lake in China. The majority of localities (94%) are scattered across the plains and low mountains (below 1,500 m a.s.l.).

### Distribution modeling

We built species distribution models to predict the ecological niches, as well as current and past ranges (during Holocene-Pleistocene) of *S. raddei*. We applied recent methodological recommendations to compute robust ecological niche models with MaxEnt 3.4.1 (Phillips, Anderson & Schapire, 2006), such as occurrence filtering, testing for distinct candidate sets of environmental variables, using multiple combinations of model parameters (features, regularization multipliers, and sets of variables), and using multiple statistical criteria for model selection.

**Figure 1:**
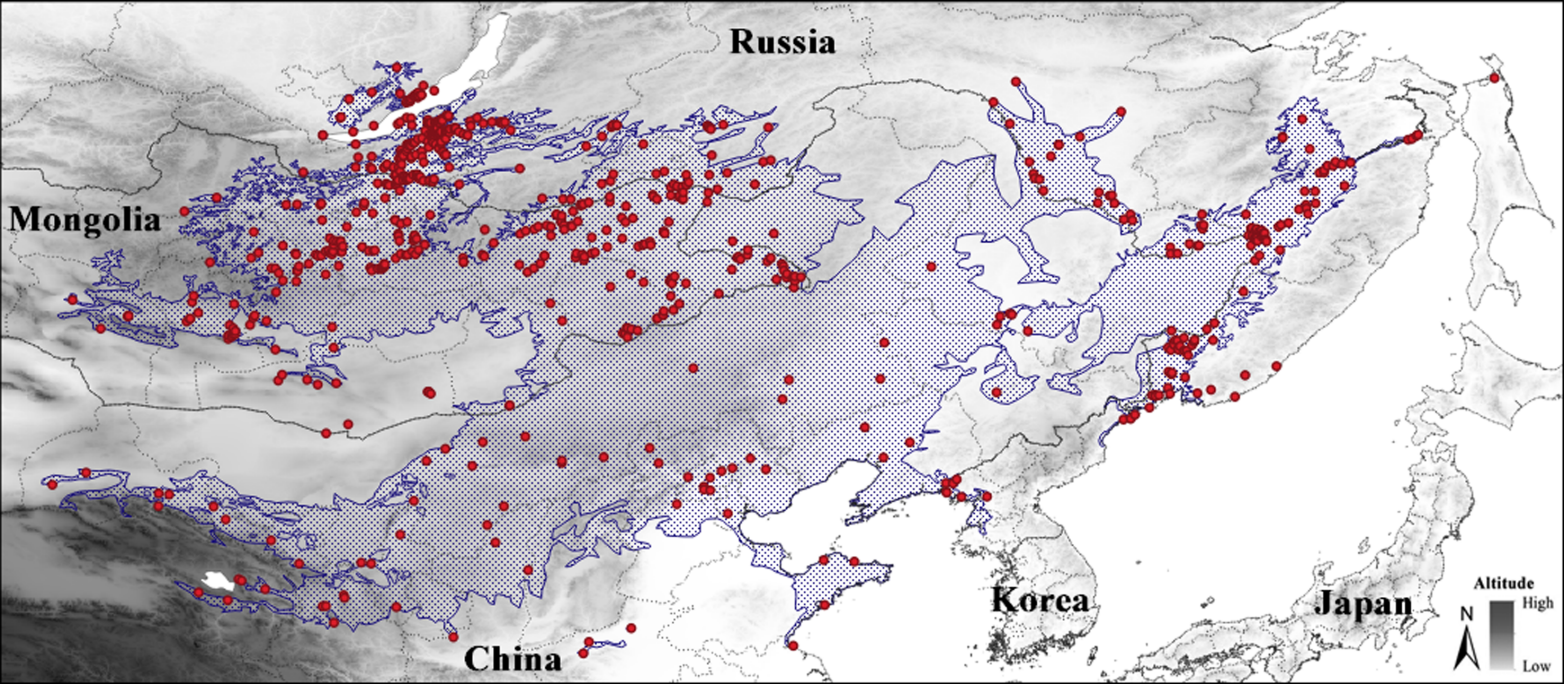
Localities of *Strauchbufo raddei* (red circles) used in the construction of ecological niche model under the current climate conditions. The limits of presumed range of the species are designated by a blue line.

A total of 714 localities with known presence of *S. raddei* were used, comprising our own records (2004–2018), museum collections and previously published data (Fig. 1; Table S1). We filtered the dataset to avoid spatial autocorrelation and duplication using NicheToolBox (Osorio-Olvera et al., 2018). We also filtered the dataset to determine the total number of localities that were at least 10 km (0.093°) apart (see Brown, 2014), which resulted in 513 presence-only locations for analyses. The number of background points was 100,000.

To compute the model under the current climatic conditions, altitude and 19 bioclimatic layers representative of the climatic data over ∼1950–2000 were extracted from the WorldClim 1.4 database (http://www.worldclim.org). Ten additional layers were considered: the aridity index (Global Aridity and Potential Evapo-Transpiration; http://www.cgiar-csi.org/data/global-aridity-and-pet-database), the global percent of tree coverage (https://github.com/globalmaps/gm_ve_v1), and eight land cover variables (spatial homogeneity of global habitat, broadleaf forests, needleleaf forests, mixed forests, shrubs, barren, herbaceous and cultivated vegetation; https://www.earthenv.org/). To consider topography in the model, four landscape layers were calculated with QGIS: aspect, exposition, slope, and terrain roughness index. These layers had a 30 arc seconds spatial resolution. All analyses were conducted under the WGS 84 projection with a species-specific mask covering to the area of occurrence of the species, as well as adjacent regions (from 25° to 65°N and 56° to 146°E).

To eliminate predictor collinearity before generating the model, we calculated Pearsons’s correlation coefficients for all pairs of bioclimatic variables using ENMTools (Warren, Glor & Turelli, 2010). For correlated pairs (—r—>0.75), we excluded the variable that appeared the least biologically important for *S. raddei*. The resulting dataset contained eight bioclimatic variables: Bio 1 (annual mean temperature; °C ×10), Bio 2 (mean diurnal range; °C ×10), Bio 3 (isothermality; Bio2/Bio 7 ×100), Bio 4 (temperature seasonality; CV ×100), Bio 5 (maximum temperature of warmest month; °C ×10), Bio 15 (precipitation seasonality; CV), Bio 16 (precipitation of wettest quarter; mm), and Bio 19 (precipitation of coldest quarter; mm). We then applied a jackknife analysis to estimate the relative contributions of variables to the MaxEnt model.

**Figure 2:**
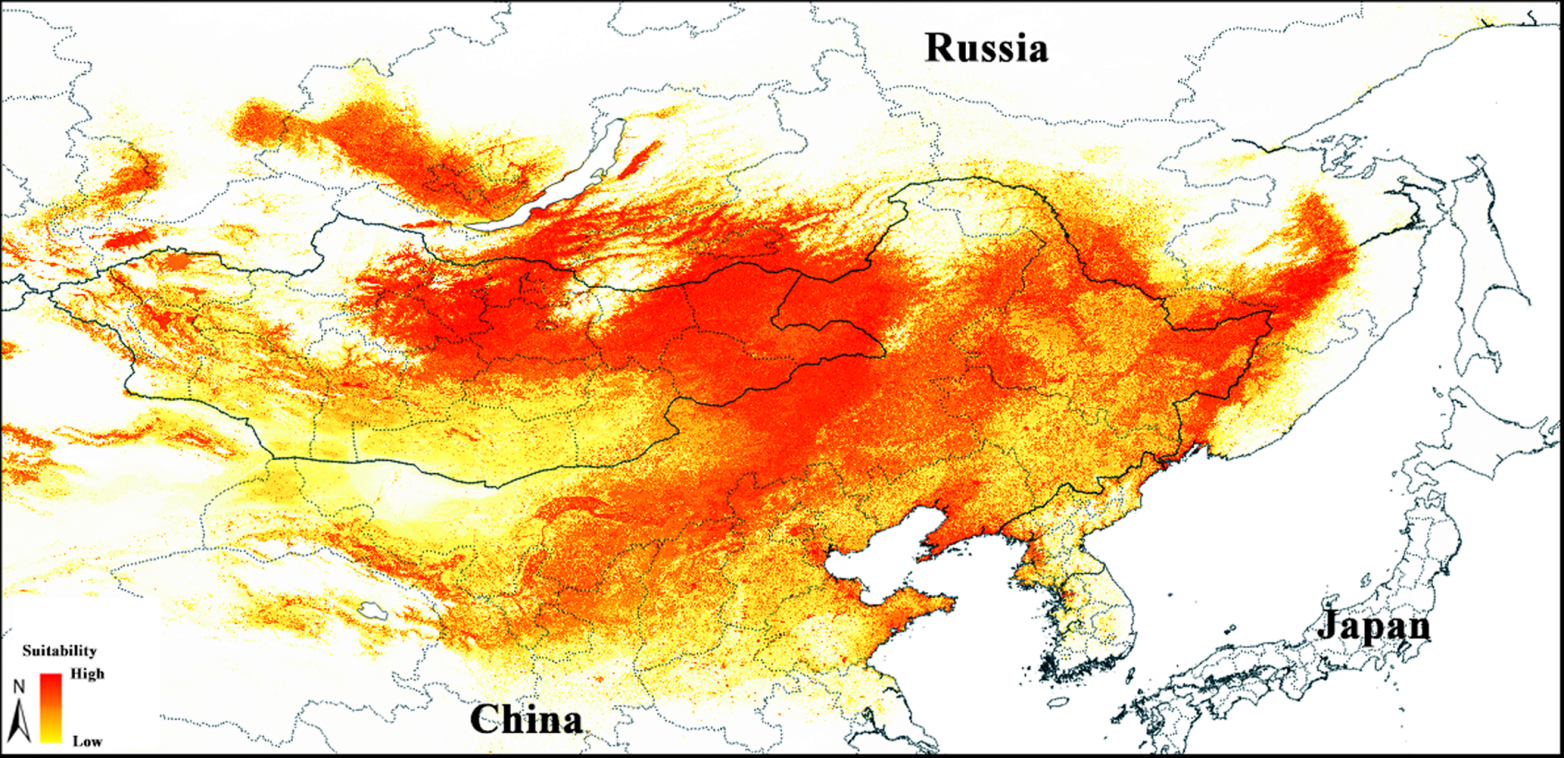
Median prediction of suitable regions for *Strauchbufo raddei* distribution under the current climate conditions.

**Figure 3:**
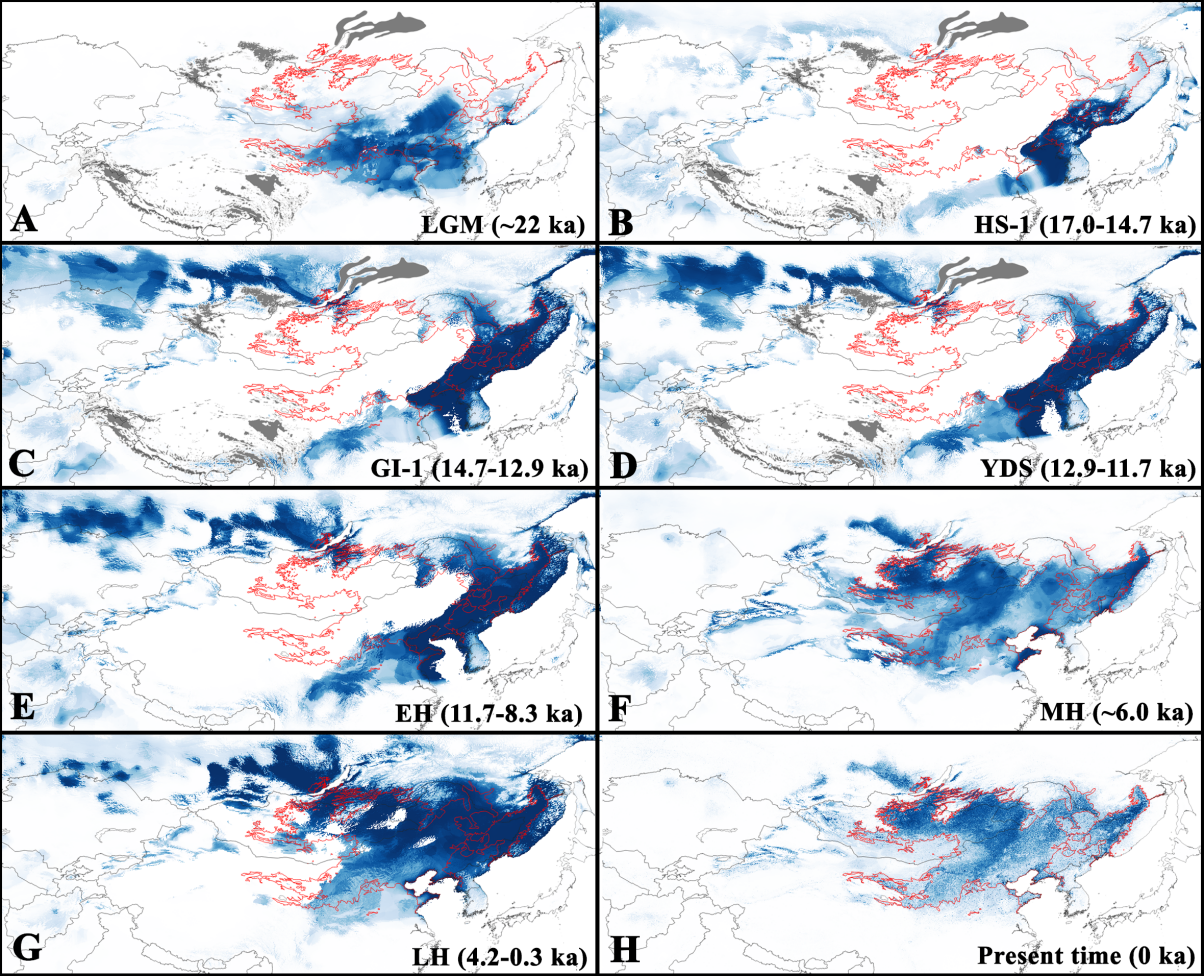
Predicted suitable regions for *Strauchbufo raddei* distribution under the Holocene and Late Pleistocene climate conditions. (A) Last Glacial Maximum (LGM); (B) Heinrich Stadial 1 (HS-1); (C) Greenland Interstade 1 (GI-1); (D) Younger Dryas Stadial (YDS); (E) Early Holocene (EH); (F) Mid Holocene (MH); (G) Late Holocene (LH); and (H) present time. Territories covered by glaciers (according to Becker et al., 2015) are in grey. Limits of current distributional range are designated by red line. The intensity of blue color relates to degree of habitat suitability (dark is high suitable and light is little suitable) for the species.

**Figure 4:**
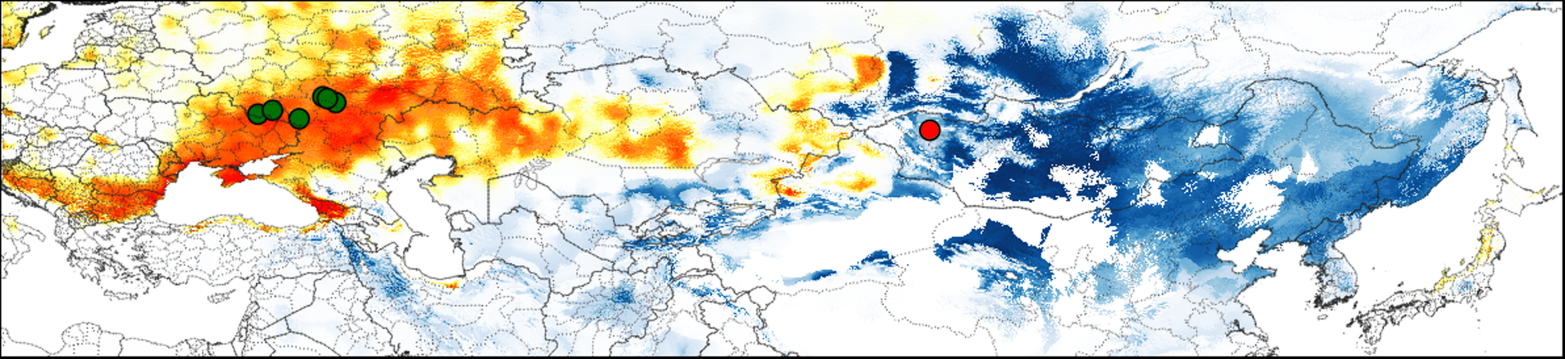
Predicted suitable regions for *Strauchbufo raddei* distribution under the mid-Pleistocene climate conditions. The model constructed using the European fossil records is in orange. The projection of the ecological niche of *S. raddei* using climatic conditions during the mid-Pleistocene is in blue. Locations of the Pleistocene (800–700 ka) fossil records of *S. raddei* in Europe and unspecified toads in Mongolia are designated by green and red cycles, respectively.

We ran the MaxEnt program for ten bootstrapped replicates with 20% random test percentage testing. Model calibration consisted in the evaluation of models created with distinct regularization multipliers (0.5 to 6 at intervals of 0.5), feature classes (resulted from all combinations of linear, quadratic, product, threshold, and hinge response types), and from two different sets of layers. The first set consisted of all 23 layers. The second was restricted to the four most valuable layers (Bio 1, Bio 4, Bio 15, and Bio 19). The best parameter settings were selected considering statistical significance (partial ROC), predictive power (omission rates E = 5%), and complexity level (AICc) obtained using the R package kuenm (Cobos et al., 2019). Additionally, the model performance was evaluated using the Area Under the Curve (AUC; ranging 0–1) and the True Skill Statistic (TSS; ranging from −1 to +1) of the 10 percentile training omission threshold (Allouche, Tsoar & Kadmon, 2006). The ClogLog output format (ranging 0–1) was chosen for processing resulting maps (Phillips et al., 2017). Figures 1–4 were plotted using QGIS v.3.10.1 software.

To project the current ecological niche of *S. raddei* on climate conditions during the Holocene and Late Pleistocene, we applied eight sets of bioclimatic layers (Table S2) with species-specific mask. Additionally, to compute two Pleistocene models, based on (1) the fossil records dating from 800–700 ka and (2) the projections of the current ecological niche of *S. raddei* on climate conditions of the Middle Pleistocene (around 787 ka), we used masks covering the area of current and past distributions of the species (from 30° to 60°N and 15° to 146°E).

Niche overlaps were estimated using D distance (Schoener, 1968) in ENMTools with niche similarity quantified statistically from 0 (no overlap) to 1 (identical niche models) based on potential niche models of the species. Data from environmental layers were extracted using the QGIS Point Sampling Tool Plugin (https://plugins.qgis.org/plugins/pointsamplingtool/). We applied the one-way ANOVA for post-hoc comparison of means (Sheffe test) in Statistica 6.0.

## Results

### Distribution modeling of species under the current environmental conditions

To calibrate the model, we assessed 696 replicates (Table S4), all of which were statistically significant when compared with a null model of random prediction. Of these significant models, 526 (76%) met the omission criterion of 5%. Four (0.6%) were statistically significant and met the AICc criteria. Only a single model was statistically significant among models meeting both omission rate and AICc criteria. Performance metrics for parameter settings used for creating this final model are given in Table S3. This model was created with the first set of environmental layers (*n* = 23), regularization multiplier 1.5, and all five response types of feature classes. Of the parameters included in the model, precipitation seasonality, annual mean temperature, temperature seasonality, and precipitation of coldest quarter were the variables with the highest percentage contributions (21%, 17%, 15%, and 11% respectively; Table S4). Other parameters had no notable contribution (equal or less than 7%). The average AUC and TSS evaluations were 0.962 (SD = 0.002) and 0.814 (SD = 0.009) indicating a high predictive power of the final model.

The median of the selected model identified areas with different levels of suitability for *S. raddei* across open landscapes of the Eastern Palearctic (Fig. 2). Highly suitable areas were concentrated in southern Siberia (Pre- and Trans-Baikal), northern and western Mongolia, Northern and Northeastern China and adjacent territories of the Russian Far East. The suitability declined towards southern Mongolia, as well as Northwest and East of China. The lower part of the Amur River valley and Sakhalin Island were characterized by little suitable environmental conditions. Some areas in southern Siberia (Irkutskaya Oblast, Barguzin and North-Baikal valleys in Buryatia, lowlands in republics of Khakassia and Tuva), western Mongolia and adjacent China, where *S. raddei* is currently absent, displayed suitable habitat for *S. raddei* as well.

### Distribution of species at the Holocene-Late Pleistocene

Fossil records of *S. raddei* dating back to the end of the Late Pleistocene (14–11 ka) are located in the Buryatia Republic of Russia only (Table S5). The projection of the current ecological niche for *S. raddei* under the Holocene and Late Pleistocene climatic conditions allowed us to consistently analyze shifts in range boundaries during the period studies. During the Last Glacial Maximum (about 22 ka) the presumed range of *S. raddei* covered a relatively large territory, mostly coinciding with the southern range of the species’ current distribution in China and North Korea (Fig. 3). Also, the species was presumably distributed in the area now covered by the Yellow Sea and penetrated to the south of current Mongolia and southernmost Primorsky Krai (Russia).

During the warmer Heinrich Stadial 1 (HS-1; 17.0–14.7 ka), the range limits of *S. raddei* strongly changed (Fig. 3). In China, it shifted to the south. Besides, the highly suitable territories were concentrated on the drainage area that is now covered by the Yellow Sea, the adjacent Korean, Shandong and Liaodong peninsulas, southern Manchuria (China) and Primorsky Krai (Russia).

During the Greenland Interstade 1 (GI-1; 14.7–12.9 ka) and the Younger Dryas Stadial (YDS; 12.9–11.7 ka), the distribution limit of *S* *. raddei* in China did not greatly change (Fig. 3). However, the species greatly expanded north, fully restoring the current species range in the Russian Far East. During this period the species was also able to disperse to the Sakhalin Island (Russia). However, the sea level began to rise gradually, occupying the species range in the area of the Yellow Sea.

Since the Early Holocene (11.7–8.3 ka), the distribution of the species in China shifted to the north, reaching its current distribution. Additionally, along the Amur River valley the species penetrated into the Baikal Region of Siberia (Fig. 3). During the Mid and Late Holocene, *S . raddei* occupied vast dry territories in Northeastern China and Mongolia.

### Distribution of the species during the Günz glacial stage

Several fossil records of *S. raddei* dating from the Günz glacial stage in the Pleistocene (800–700 ka) were found in the European part of Russia (Fig. 4; Table S5). In Asia, fossil remains of toads which could belong to *S. raddei*, were collected near Khyargas Lake in Western Mongolia (Fig. 4; Table S5). Additional two Asian records of *S. raddei* (Table S6) were found in the vicinities of Beijing in China and in Dodo-Gol River in Buryatia (Russia), but they can belong to the later periods of the mid-Pleistocene and therefore did not used in our analyses.

The projection of current ecological niche of *S. raddei* on climate conditions at the start of the mid-Pleistocene (around 787 ka) allowed us to estimate distributional limits of the species at that period. The presumed range of the species (Fig. 4) covered a huge territory in Eastern Asia extending to Khyargas Lake in Mongolia, but didn’t penetrate to Europe.

To compute a corresponding species model, we used six European localities of *S. raddei* dating from 800–700 ka (Table S6 ; nos. 10–15) and bioclimatic layers reflecting the mid-Pleistocene climate conditions (around 787 ka). The median of this model identified highly suitable areas in some regions of Europe: the Danube River valley, the Crimea, the western Caucasus, Kazakhstan, and the southern part of East-European Plain.

**Figure 5:**
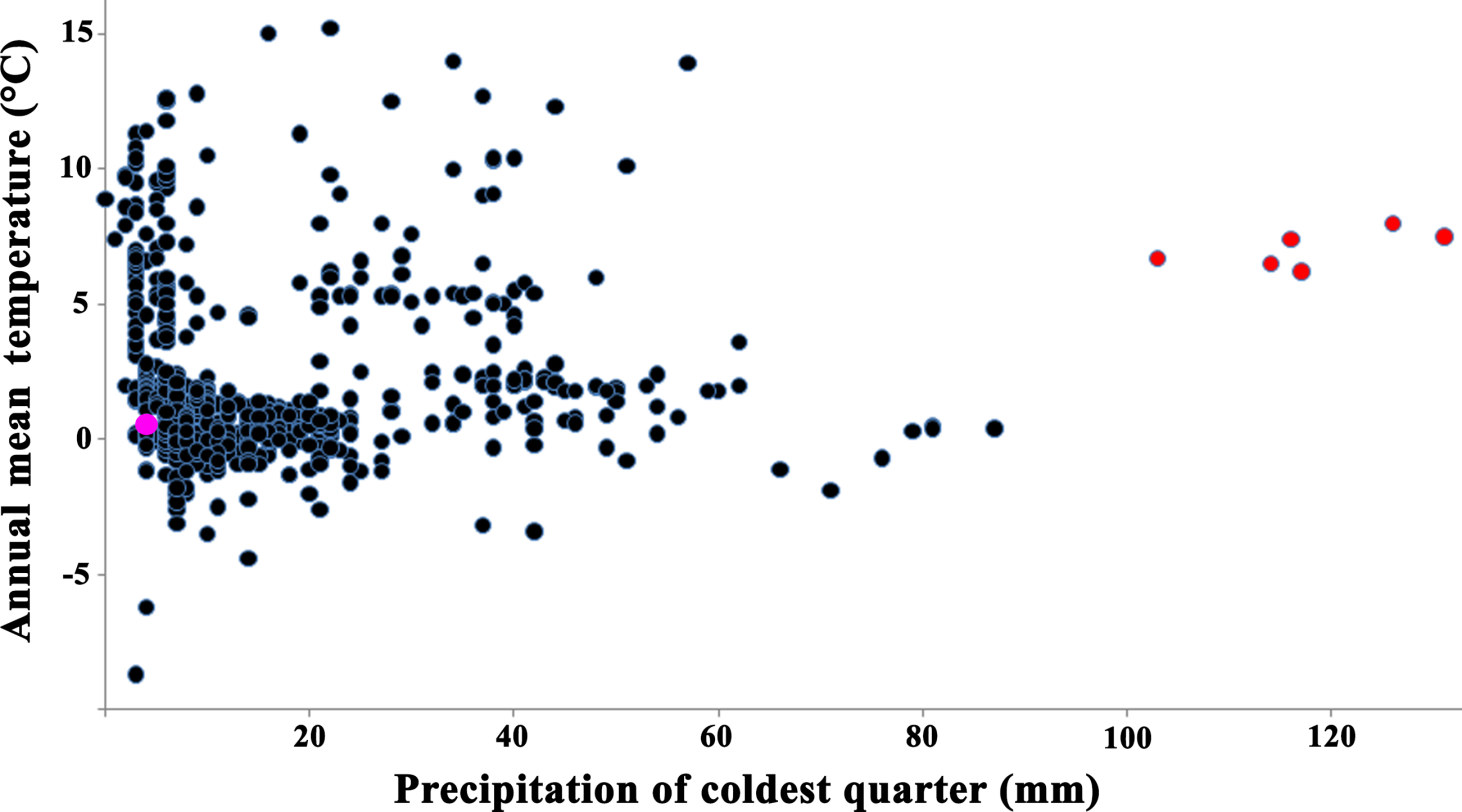
Relationships between annual mean temperature and precipitation of coldest quarter for localities of *Strauchbufo raddei*. Current time localities are designated by black circles; the Günz glacial stage fossil records of the species in Europe are red circles and unspecified toads in Mongolia is a rose circle.

These two mid-Pleistocene models intersected weakly with each other (D distance = 0.22). The majority of climatic parameters (79%) for the current distribution of *S. raddei* and the European Pleistocene fossil records were significantly different (Table S7). The climatic condition in which the species lives today is colder and drier (with little snow in winter) than it was in Europe during the mid-Pleistocene (Fig. 5).

## Discussion

Our results in conjunction with previously published data allowed us to propose a hypothesis explaining the formation of the current species range. Based on molecular data (Van Bocxlaer et al., 2010; Peng et al., 2015; Portik & Papenfuss, 2015; Liedtke et al., 2016), the origin of the genus *Strauchbufo* could be estimated as the Early Miocene-Eocene (about 21.0–39.5 Ma). The closest relatives are genera *Bufo*, *Bufotes*, *Epidalea* and *Sabahphrynus*, which are distributed throughout Eurasia. The place of origin of *Strauchbufo* could be Asia, because all recently known earliest fossil records (from the Miocene) were found in Kazakhstan, eastern China and the Baikal region of Siberia (Table S5).

During the Pliocene, the species begins to be found not only in Asia, but also in the current territory of Ukraine and European Russia (Table S5–S6). Based on paleontological data, in Eastern Europe the species existed until the end of the mid-Pleistocene (380–130 ka), when it became extinct due to the change of climatic conditions and/or displacement by green toads (genus *Bufotes*), which are characterized by very close ecological requirements (Ratnikov, 2009). Our data show that the ecological niche of the European representatives of *Strauchbufo* was very different from the modern one and probably different from that of Asian populations during the mid-Pleistocene. Perhaps, this indicates the beginning of divergence between these groups of populations.

The fossil records of the species for the Late Pleistocene are known from the territory of Mongolia and Buryatia only (Table S5). It is likely that during this period the range boundaries of this toad changed depending on climate fluctuations. Molecular evidences (Dong et al., 2012) can complement data about the distribution dynamics of Asian populations of *S. raddei* during the Middle and Late Pleistocene. Genealogical reconstructions detected two major western and eastern lineages that diverged during the Pliocene (about 2.1 Ma), and the eastern lineage can be subdivided into central and eastern sublineages. At the end of the Middle and Late Pleistocene, population sizes from all lineages increased and then plateaued during the last glacial maximum (around 22 ka), followed by another rapid growth phase.

According to our analysis, during the last glacial maximum (about 22 ka) refugia of the species were located in the territory of current China and North Korea (Fig. 3). Changes in climate during the Heinrich Stadial 1 (17.0–14.7 ka) led to the displacement of populations belonging to the western and central lineages to the south. At this period the eastern lineage seems to have inhabited the Liaodong and Korean peninsulas, the area now covered by the Yellow Sea, as well as the southern part of Primorsky Krai (Russia). Since the end of the Pleistocene (14.7–12.9 ka), populations belonging to the lineage greatly expanded to the north of the Russian Far East and later in Siberia, Mongolia and northeastern China.

Thus, our data support the hypothesis (Dong et al., 2012) of a remarkable dispersal pattern by the eastern lineage at the end of the Pleistocene and Holocene. The adaptation of *S. raddei* to cold temperatures and desert habitats might have permitted the rapid colonization of new areas and promoted population expansions.

## Conclusions

The influence of the Pleistocene climate fluctuations on changes in fauna of East Asia is still poorly studied. Therefore, we analyzed changes in distribution of a widespread amphibian species, the Mongolian toad, since the Late Pleistocene using environmental modeling and involving paleontological and genetic data. Such combined methods allowed us to study this process in details. We found that at the end of the Pleistocene, the range of the toad was most severely reduced during the Heinrich Stadial 1. During the stadial, the main range of the species had strongly shifted to the south, covering a vast territory outside the modern range. Only during the Middle Holocene, the southern border of the species range moved back to the north leaving only rare isolated populations in this refugial region. The main route of postglacial dispersal of the species to the north was Eastern Manchuria (China) and the Russian Far East, and then the Amur River Valley. Perhaps, this pattern coincides with postglacial routes of dispersal for most amphibian and other terrestrial vertebrate species inhabiting this region but this topic is poorly studied. We have established that the species dispersed to the northern region of the Sakhalin Island at the end of the Pleistocene. Additionally, we revealed that after dispersing to new European territories during the Pliocene, the species strongly changed its own environmental requirements giving rise to a new ecologically distinct form that went extinct at the end of the Middle Pleistocene.

##  Supplemental Information

10.7717/peerj.9216/supp-1 Table S1 Occurrence data used to build the species distribution models; mapped in Fig. 1

10.7717/peerj.9216/supp-2 Table S2 Bioclimatic layers used to compute the GIS models under the Holocene and Pleistocene climate conditions

10.7717/peerj.9216/supp-3 Table S3 Performance statistics for all candidate models Note: l is linear, q is quadratic, p is product, t is threshold, and h is hinge.

10.7717/peerj.9216/supp-4 Table S4 Performance metrics for parameter settings used for creating the present time model and relative contribution (%) of variables

10.7717/peerj.9216/supp-5 Table S5 Fossil records of Strauchbufo species in Asia

10.7717/peerj.9216/supp-6 Table S6 Fossil records of Strauchbufo raddei in Europe

10.7717/peerj.9216/supp-7 Table S7 The climatic parameters for localities of Strauchbufo raddei Present time localities (*n* = 713). Pleistocene (Europe) is the mid-Pleistocene European fossil records (*n* = 6). Pleistocene (Mongolia) is undetermined toads from Mongolia (*n* = 1). Significant differences (*p* < 0.05) between current and the mid-Pleistocene (for European fossil records) climatic parameters are underlined.

## Additional Information and Declarations

### Data Availability

The following information was supplied regarding data availability: The raw data and data resulting from analyses are available in the Supplemental Tables and figure files.
