# Supplementary material for: Reconstruction of past distribution for the Mongolian toad, Strauchbufo raddei (Anura: Bufonidae) using environmental modeling
Source: PeerJ. 2020 Jun 5;8:e9216. doi: 10.7717/peerj.9216 (PMC7278888; doi:10.7717/peerj.9216)
Supplement: Table S4 [file peerj-08-9216-s004.pdf]

Table S4. Performance metrics for parameter settings used for creating the present time model and relative contribution (%) of variables.

**Variable**	**Value**
Partial ROC	0
Omission rate 5%	0.039
AICc	12685
Delta AICc	0
Annual mean temperature (Bio1)	16.6
Mean diurnal range (Bio 2)	0.5
Isothermality (Bio 3)	2.0
Temperature seasonality (Bio 4)	14.8
Maximum temperature of warmest month (Bio 5)	7.2
Precipitation seasonality (Bio 15)	20.9
Precipitation of wettest quarter (Bio 16)	1.8
Precipitation of coldest quarter (Bio 19)	11.0
Broadleaf forest	0.6
Needleleaf forest	0.7
Mixed forest	1.5
Shrubs	0.9
Barren	3.0
Herbaceous vegetation	5.1
Cultivated vegetation	3.0
Altitude	2.4
Aridity index	1.0
Aspect	0.2
Exposition	0.1
Habitat homogeneity	1.7
Slope	0.7
Terrain roughness index	0.3
Tree coverage percent	3.9
