# Supplementary material for: Reconstruction of past distribution for the Mongolian toad, Strauchbufo raddei (Anura: Bufonidae) using environmental modeling
Source: PeerJ. 2020 Jun 5;8:e9216. doi: 10.7717/peerj.9216 (PMC7278888; doi:10.7717/peerj.9216)
Supplement: Table S7 — Present time localities (n = 713). Pleistocene (Europe) is the mid-Pleistocene European fossil records (n = 6). Pleistocene (Mongolia) is undetermined toads from Mongolia (n = 1). Significant differences (p < 0.05) between current and the mid-Pleistocene (for European fossil records) climatic parameters are underlined. [file peerj-08-9216-s007.pdf]

Table S7. The climatic parameters for localities of *Strauchbufo raddei*. Present time localities (n = 713). Pleistocene (Europe) is the mid-Pleistocene European fossil records (n = 6)**.** Pleistocene (Mongolia) is undetermined toads from Mongolia (n = 1). Significant differences ($p < 0.05$) between current and the mid-Pleistocene (for European fossil records) climatic parameters are underlined.

**Bioclimatic variables**	**Present time**		**Pleistocene (Europe)**		**Pleistocene (Mongolia)**	**Sheffe test (df = 717)**	
	**Mean ± SD**	**Range**	**Mean ± SD**	**Range**		**p-value**	**Error (MS)**
Annual mean temperature (°C)	2.0 ± 3.1	-8.7-15.2	7.1 ± 0.7	6.2-8.0	0.6	0.00007	944.5
Temperature seasonality (CV)	13668 ± 1418	8343-17053	12395 ± 552	11602-13024	18574	0.02827	1999000.0
Mean temperature of wettest quarter (°C)	19.1 ± 2.4	3.2-26.7	21.4 ± 0.5	20.8-22.2	23.4	0.02069	589.6
Mean temperature of driest quarter (°C)	-14.8 ± 4.7	-23.9-1.4	-2.3 ± 2.1	-5.1-0.3	-17.8	0	2199.5
Mean temperature of warmest quarter (°C)	19.6 ± 2.3	3.2-27.4	22.9 ± 0.5	22.3-23.7	23.4	0.00072	529.6
Mean temperature of coldest quarter (°C)	-17.8 ± 4.6	-27.1-1.4	-7.0 ± 1.3	-8.3--5.5	-22.0	0	2116.6
Annual precipitation (mm)	346 ± 191	41-1299	531 ± 39	478-596	112	0.01780	36088.0
Precipitation of wettest month (mm)	88 ± 42	14-393	55 ± 4	50-61	29	0.05394	1777.3
Precipitation of driest month (mm)	4 ± 4	0-22	25 ± 3	21-30	0	0	13.8
Precipitation seasonality (CV)	103 ± 15	41-132	21 ± 1	20-23	89	0	237.7
Precipitation of wettest quarter (mm)	243 ± 118	35-1031	155 ± 12	142-177	68	0.06947	13962.0
Precipitation of driest quarter (mm)	12 ± 12	0-67	91 ± 8	81-104	2	0	140.2
Precipitation of warmest quarter (mm)	228 ± 114	35-1031	150 ± 13	137-173	68	0.09150	12837.0
Precipitation of coldest quarter (mm)	16 ± 14	0-87	118 ± 10	103-131	4	0	200.4
